# Nitrogen Fertilizer Levels Affect the Growth and Quality Parameters of *Astragalus mongolica*

**DOI:** 10.3390/molecules25020381

**Published:** 2020-01-16

**Authors:** Lingling Wang, Lucun Yang, Feng Xiong, Xiuqing Nie, Changbin Li, Yuanming Xiao, Guoying Zhou

**Affiliations:** 1 Key Laboratory of Tibetan Medicine Research, Northwest Institute of Plateau Biology, Chinese Academy of Sciences, Xining 810008, China; m15565477168@163.com (L.W.); yanglucun@nwipb.ac.cn (L.Y.); 18097203196@163.com (F.X.); niexiuqing123@163.com (X.N.); lichangbin900912@126.com (C.L.); xiaoyuanming16@163.com (Y.X.); 2 Qinghai Key Laboratory of Qinghai-Tibet Plateau Biological Resources, Xining 810008, China; 3 University of Chinese Academy of Sciences, Beijing 100049, China

**Keywords:** nitrogen fertilizer, Astragaloside IV, mullein isoflavone glucoside, harvest time, yield, Qinghai-Tibet Plateau

## Abstract

Owing to overexploitation, wild resources of *Astragalus mongolica*, a Chinese herbal plant that is widely distributed in the arid and semi-arid areas of Northern China, have gradually become exhausted, and therefore, commercial cultivation is increasingly important to meet the growing demand for astragalus and reduce the pressure on wild populations. Nitrogen level is an important factor that affects the yield and quality of *A. mongolica*. However, uniform standards for fertilization among production areas have not yet been determined. In this study, the effect of nitrogen fertilizer treatment on the yield and quality of *A. mongolica* in the Qinghai-Tibet Plateau was explored using a control treatment (no added nitrogen, N0) and five different nutrient levels: 37.5 kg/ha (N1), 75 kg/ha (N2), 112.5 kg/ha (N3), 150 kg/ha (N4), and 187.5 kg/ha (N5). According to grey relational analysis, the optimal nitrogen fertilizer treatment was the N4 level followed by the N5 and N2 levels. Nitrogen fertilizer significantly increased the root biomass, plant height, root length, and root diameter. However, nitrogen fertilization had no significant effect on the content of Astragaloside IV and mullein isoflavone glucoside. The content of ononin and calycosin continually accumulated throughout the growing period. The results showed that the ononin and calycosin content under N4 and N2 is higher than other levels and there is not significantly different between different nitrogen fertilizer levels about them. The content of formononetin decreased gradually with the progression of the growing season. The optimal nitrogen fertilizer treatment for *A. mongolica* is recommended to be 150 kg/ha and the content of active compounds and yield were observed to reach the maximum in October.

## 1. Introduction

*Astragalus mongolica* (*A. mongolica*) is a multipurpose leguminous herb that has been widely used in China for thousands of years as a medicinal and food plant because of its remarkable pharmacological efficacy [1]. The plant, which was medically used by its root, is cultivated in drought conditions with cool weather and fertile soil. The farmer breeds seedlings in the first year, and plants and harvests it in the second year. This species is also used in the textile and pharmaceutical industries as an industrial raw material. Owing to the toughness and preservation of astragalus fiber, the practicability and effectiveness of textiles made from it are greatly improved [2]. In recent years, the medicinal value of the root of astragalus has been continuously examined in the pharmaceutical industry and its use has become increasingly more popular, not only locally in China but also abroad, particularly in Southeast Asian countries such as in Korea, Japan, and Singapore [3]. 

With effects such as reinforcing Qi, assisting detumescence, and reducing the sweat function, *A. mongolica* was officially listed in the 2015 edition of Chinese Pharmacopoeia [4]. Many modern pharmacological studies have shown that *A. mongolica* possesses anti-inflammatory [5], anti-oxidation, and anti-tumor [6] effects, and also influences the immune system [7]. Its ononin, calycosin, and formononetin components serve to reduce blood pressure [8] and sugar concentration, and have neuroprotective [9] and liver protection functions [10]. People often use the root as a decoction, soup in the diet, or decoction pieces in the boiled water to perform the medicinal effect. These excellent pharmacological benefits are attributed to its active components, including flavonoids, saponins, polysaccharides, and other compounds. The Chinese Pharmacopoeia defined two of these components as quality control indexes, namely mullein isoflavone glucoside and Astragaloside IV, with content levels no less than 0.02% and 0.04%, respectively [4]. Similarly, the European Pharmacopoeia stipulated that the content of Astragaloside IV should not be less than 0.04% [11]. Meanwhile, the Japanese Pharmacopoeia does not require the content for ingredients [12] and studies have assessed ononin, calycosin, and formononetin as part of the quality evaluation criteria [13]. 

*Astragalus mongolica* is mainly distributed in the arid or semi-arid areas of northern China, such as Gansu and Shanxi Provinces in China [14]. They have been crude processed after harvesting and then enter the market. The better the appearance, such as long and thick root, higher the market price. The yield, on the other hand, is positively correlated with income for farmers. With the wild resources sharply diminishing and the constant growth of market demands, cultivation has been considered a viable mean to reduce the pressure on natural populations. Consequently, farmers began producing *A. mongolica* using large-scale commercial cultivation methods, even as early as the 1950s [15]. However, the amount of fertilizer applied in different regions remains diversiform as no reference standard has been developed to guide farmers in this regard. Farmers often sow the urea nitrogen in the field and judge the amount by the traditional experience. Under the current and increasing demand for *A. mongolica*, the ideal fertilizer requirement for this species urgently requires clarification.

Fertilization, particularly nitrogen fertilizer [16,17], plays an extremely important role in common cultivation techniques [18,19]. Numerous studies, using experiment data on different plants, have revealed the effects of nitrogen fertilization [20]. For example, Scherer, H. W. found that the highest broad beans (*Vicia faba* L.) yields were obtained in the treatment with the lowest N-rate (100 mg N/pot) and in the treatment with the highest N rate (800 mg N/pot) [21]; Emnova, E. E. et al. reported that soybean (*Glycine max* L.) production was positively affected by urea [22]. Achakzai, A. K. K. et al. concluded that different fertilizer levels significantly (*p* < 0.05) influenced most of the growth attributes of the mungbean (*Vigna radiata* (L.) *Wilczek*) [23]; and Achakzai, A. K. K. et al. got the same conclusion in the experiment of pea (*Pisum sativum* L.) [24]. Nitrogen is known to be functional in the construction of amino acids and chlorophyll, which can influence plant growth and development by affecting photosynthesis and the uptake of minerals [25,26]. Although numerous studies have been conducted to assess the effects of nitrogen fertilizer on *A. mongolica*, most research attention has been focused on geo-authentic habitats distributed at low altitudes, and the conclusions of these studies are inconsistent and confusing. For example, Jing reported that nitrogen fertilization had the best comprehensive benefit when applied at 30 and 60 kg/ha in the Gansu Province in China [27]. In the Shanxi Province in China, Cheng considered the yield and active ingredients of *A. mongolica* and determined the optimal nitrogen application rate to be 150 kg/ha [28], while in another experiment conducted by Gao [29], the optimal amount of nitrogen applied was found to be 225 kg/ha. To date, no study has determined and reported a standard for the amount of nitrogen fertilizer to be applied to different areas and little research has been conducted regarding this aspect in the Qinghai-Tibet Plateau. As the “third pole” of the world, the ecological environment of Qinghai-Tibet Plateau has a great influence on crop growth [30]. For instance, the average temperature of the Qinghai-Tibet Plateau is generally 5 °C colder than other areas at the same latitude, thus, the growth period of *wheat* (*Triticum aestivum* L.) is prolonged and the harvest time is postponed [31]. Harvest time is also important for the yield and quality of *A. mongolica*. Gao showed that the content of secondary metabolites in *A. mongolica* differed among different harvest periods and that a higher amount of growth indicators and secondary metabolites accumulated in late September and early October [29].

As a result, there is a hypothesis that not only the nitrogen levels, but also harvesting times influence the growth and active compounds of *A. mongolica*. The purpose of the present study is to examine how different levels of nitrogen fertilizer affect the growth and active compounds of *A. mongolica* and to confirm which month is the best for harvesting. Specifically, the plant height, root length, root diameter, and the fresh and dry weight of aboveground parts and roots were measured. The flavonoids and Astragaloside IV content were also determined by reverse phase HPLC. The optimal nitrogen level was determined using GRA.

## 2. Results

### 2.1. Effect of Nitrogen Fertilizer on the Biomass and Growth Traits of A. mongolica 

**Figure 1:**
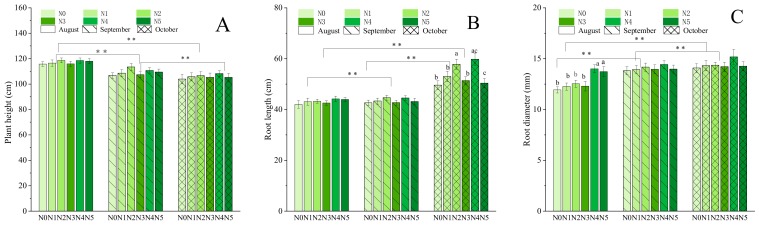
Plant height (cm), root length (cm), and root diameter (mm) in response to different nitrogen levels: 0 kg/ha (N0, control), 37.5 kg/ha (N1), 75 kg/ha (N2), 112.5 kg/ha (N3), 150 kg/ha (N4), and 187.5 kg/ha (N5), and harvest stages from August to October. (**A**) Plant height from August to October; (**B**) root length from August to October; (**C**) root diameter from August to October, respectively. Significant differences at *p* < 0.05 are indicated by different letters. Significant differences at *p* < 0.01 are indicated by ** between months.

The data presented in Figure 1 suggest that plant height (cm/plant), root length (cm/plant), and root diameter (mm/plant) were influenced by the harvesting times and the nitrogen fertilizer has influence on the root length in October and root diameter in August. The natural plant height decreased gradually with the delay in the harvesting time because of the mature of seeds. The highest plant height was observed in the N4 treatment group, which peaked in August, and significant differences were observed between the five levels of nitrogen fertilizer. The maximum plant heights also peaked in August for the other groups, except for the N2 and N4 treatment groups, in which the maximum plant heights peaked in September and October, respectively. In the latter harvest stage, the plant heights decreased by 10.16%, 9.18%, 10.07%, 9.23%, 8.45%, and 10.83% compared with August in the N0, N1, N2, N3, N4, and N5 treatment groups, respectively. The Figure 1 shows that the plant heights differed significantly among months (*p* < 0.01). 

The root lengths and root diameters showed some regularity among treatments, whereby maximum values were observed in the N2 or N4 treatment groups throughout the experimental period. The root lengths and diameters increased gradually with the increase in cultivation time and reached a maximum in October. The N4 dose caused a significant difference in these two indicators, whereby the longest root (59.76 cm) was obtained in October compared with August (44.13 cm) and September (44.54 cm), with increases of 35.41% and 34.17%, respectively. The root diameters ranged from 11.93 to 15.17 mm, and reached a maximum in October, with incremental increases of 12.56% and 2.50% compared with the previous 2 months, and no significant differences were observed among the nitrogen fertilizer treatment groups in September and October. As shown in Figure 1, the root length and root diameter indexes differed significantly among months (*p* < 0.01).

**Figure 2:**
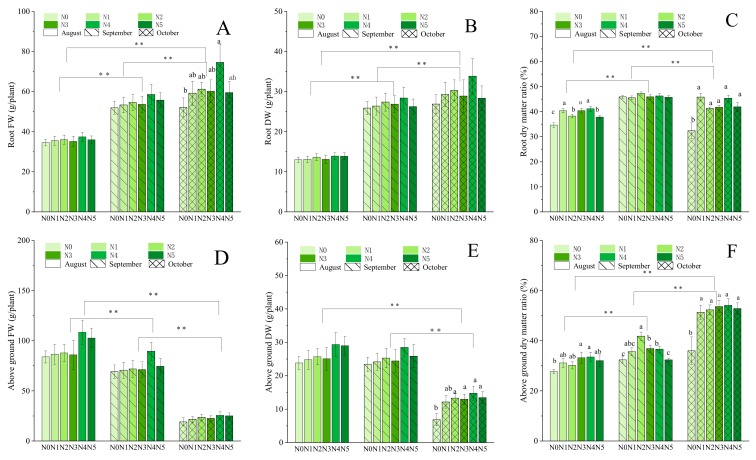
Fresh weight (Fw, g/plant), dry weight (Dw, g/plant), and dry matter ratio (%) of roots and aboveground parts in response to different nitrogen levels: 0 kg/ha (N0, control), 37.5 kg/ha (N1), 75 kg/ha (N2), 112.5 kg/ha (N3), 150 kg/ha (N4), and 187.5 kg/ha (N5), and harvest stages from August to October. (**A**) Root Fw from August to October; (**B**) root Dw from August to October; (**C**) root dry matter ratio from August to October; (**D**) aboveground Fw from August to October; (**E**) aboveground Dw from August to October; (**F**) aboveground dry matter ratio from August to October, respectively. Significant differences at *p* < 0.05 are indicated by different letters. Significant differences at *p* < 0.01 are indicated by ** between months.

As shown in Figure 2, the fresh weight and dry weight of the roots and aboveground parts revealed different growth traits. The average fresh and dry root weight in October were 52.12 and 26.92 g/plant, 59.19 and 29.29 g/plant, 61.19 and 30.30 g/plant, 60.18 and 28.88 g/plant, 74.63 and 33.85 g/plant, and 59.50 and 28.32 g/plant for the nitrogen fertilizer treatment groups N0–N5, respectively. The root fresh biomass value in the final month ranged from 52.12 g/plant to 74.63 g/plant, with incremental increases of 51.03~99.49% versus August. Meanwhile, fertilizer levels had no impact on the fresh and dry weight of the roots and aboveground parts in August and September. The ANOVA showed that the N4 traits were significantly different in root FW in October and aboveground DW in October. The root dry matter ratio showed more pronounced changes between months and differed significantly among fertilizer treatments.

On the other hand, the fresh and dry weight of the aboveground parts showed the opposite trend, i.e., decreased each month, and were highest in August and lowest in October. The biomass values of the control group (N0, without fertilization) were the lowest in all stages. The significant differences among all indicators between months are shown in Figure 2.

### 2.2. Effect of Different Levels of Nitrogen Fertilizer on the Active Compounds of A. mongolica 

**Figure 3:**
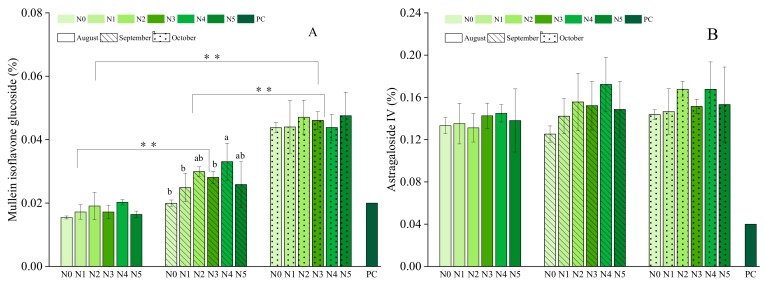
Mullein isoflavone glucoside (%) and Astragaloside IV (%) at different harvest stages from August to October ((**A**) Mullein isoflavone glucoside from August to October; (**B**) Astragaloside IV from August to October) under different nitrogen treatment levels: N0 = 0 kg/ha (unfertilized control), N1 = 37.5 kg/ha, N2 = 75 kg/ha, N3 = 112.5 kg/ha, N4 = 150 kg/ha, N5 = 187.5 kg/ha, PC = Pharmacopoeia criterion (0.02%, 0.04%). Data represent means ± standard errors (SE). Significant differences at *p* < 0.05 are indicated by different letters. Significant differences at *p* < 0.01 are indicated by ** between months.

Nitrogen fertilizer treatments had no significant effect on Astragaloside IV but had a slight significant effect on mullein isoflavone glucoside in September (Figure 3). On average, the Astragaloside IV levels were higher across the entire experimental period than specified in the Chinese Pharmacopeia (0.04%) and the values of mullein isoflavone glucoside were higher than those (0.02%) in September and October. The mullein isoflavone glucoside levels were 0.043%, 0.044%, 0.047%, 0.046%, 0.043%, and 0.047% for the N0, N1, N2, N3, N4, and N5 treatment groups, respectively, in October. The maximum mullein isoflavone glucoside content was recorded in the N5 group in October and in the N4 group in August and September. The mullein isoflavone glucoside content of *A. mongolica* was higher in all nitrogen fertilizer treatment groups compared with the control in all months and the content differed significantly from month to month. However, the differences among treatments and months of Astragaloside IV content were not significant. The highest Astragaloside IV levels were observed in the N2 treatment group in October. The Astragaloside IV levels of the plants in treatment groups N0, N1, N3, and N5 were similar, and the levels of the N2 and N4 groups were slightly higher in the last 2 months of the experiment. In August, the level in the N2 group was slightly lower than that of the control, and the levels in the N1, N3, N4, and N5 groups were higher than that of the control.

**Figure 4:**
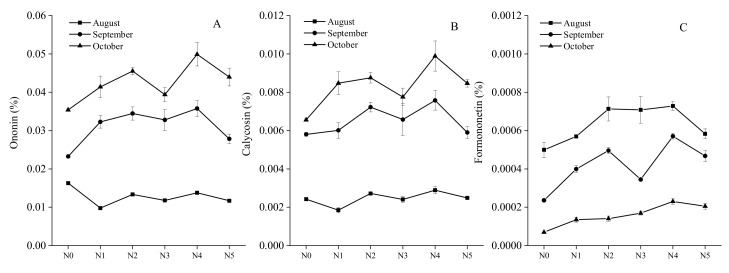
Ononin (%), calycosin (%), and formononetin (%) levels between different harvest stages from August to October ((**A**) Ononin from August to October; (**B**) calycosin from August to October; (**C**) formononetin from August to October) under different nitrogen treatment levels: N0 = 0 kg/ha (unfertilized control), N1 = 37.5 kg/ha, N2 = 75 kg/ha, N3 = 112.5 kg/ha, N4 = 150 kg/ha, N5 = 187.5 kg/ha. Data represent means ± standard errors (SE).

Although mullein isoflavone glucoside accounted for a large portion of the flavonoids, the flavonoids were comprised of other active secondary compounds that are also crucial for determining the quality (Figure 4). The content of ononin and calycosin increased each month, and reached peak levels in October, while interestingly, the average formononetin levels decreased gradually with the extension of the growing period, and very little was produced by *A. mongolica*. In October, the mean ononin levels were 0.035%, 0.041%, 0.046%, 0.039%, 0.050%, and 0.044% in fertilizer treatment groups N0–N5, respectively, i.e., 1.69 to 2.15 times higher than in August. The calycosin levels ranged from 0.0019 to 0.0099% during the 3 months and were significantly different (*p* < 0.05, ANOVA) among months. Overall, the N4 and N2 treatments resulted in better parameters compared with the other treatments. The formononetin levels recorded were 6.96 × 10^−5^%, 1.35 × 10^−4^%, 1.41 × 10^−4^%, 1.69 × 10^−4^%, 2.30 × 10^−4^%, and 2.05 × 10^−4^% for the N0, N1, N2, N3, N4, and N5 treatment groups in the final month.

### 2.3. Effect of the Different Levels of Nitrogen Fertilizer on the Yield and Quality of A. mongolica

#### 2.3.1. Effect of the Different Levels of Nitrogen Fertilizer on Yield

Yield is determined by the root fresh weight per plant and the number of plants. In the current experimental design, the nitrogen fertilizer treatments were equal with 20 × 10^4^ plants. Therefore, the yield was easy to calculate. It is believed that nitrogen fertilizer may promote an increase in the yield of *A. mongolica*. The highest yield (14,925.19 kg/ha) was obtained for the N4 dose in October compared with the control (10,423.12 kg/ha). Indeed, the level of N4 fertilizer promoted the highest outputs, i.e., 7482.80 kg/ha and 11,712.00 kg/ha in the first 2 months, respectively. The yields from the other treatments were (in descending order): 12,238.92 kg/ha, 12,035.53 kg/ha, 11,900.1 kg/ha, 11,838.24 kg/ha, and 10,423.12 kg/ha for treatments N2, N3, N5, N1, and N0, respectively. Similarly, the maximum was observed for N4 in September, and a 12.69% increase was found in this stage compared with the control. There were large fluctuations among the N0 (6901.54 kg/ha), N1 (7104.07 kg/ha), N2 (7209.33 kg/ha), N3 (7025.50 kg/ha), N4 (7482.80 kg/ha), and N5 (7190.91 kg/ha) groups in August, and the yield of the N4 group was ~8.42% higher than the control (N0).

#### 2.3.2. Comprehensive Evaluation of Quality under Different Treatments by GRA

In addition to the yield, the active compounds are also vital to evaluate the quality of *A. mongolica*. To select the most effective fertilizer treatment, the plant quality characteristics of five active ingredients and the yield were estimated by GRA.

It can be concluded from the definition of the relative correlation that the closer the value is to 1, the more correlated the treatment is with the optimal reference sequence, i.e., the better the quality. The data in Table 2 indicated that the best quality was obtained from the N4 treatment, followed by the N5, N2, N1, and N3 treatments and the worst comprehensively evaluated treatment was the blank control (N0). The value of the relative correlation of the six treatments ranged from 0.2984 to 0.5684, which revealed that the quality of medicinal materials varied greatly with different nitrogen applications. The comprehensive quality of *A. mongolica* was the best under the N4 (150 kg/ha) nitrogen fertilizer treatment. 

## 3. Discussion

*A. mongolica* is widely used in many fields and thus has great economic viability, however, a sustainable supply is the key to its marketability and profitability, of which quality is a top priority. In the present study, five active compounds, i.e., Astragaloside IV, mullein isoflavone glucoside, ononin, calycosin, and formononetin, were tested to assess the effects of different nitrogen fertilizer treatments on the quality of *A. mongolica*. This practice is consistent with previous studies, which determine the best fertilizer amounts by active compositions [13,32]. To some extent, the quantity is also part of the evaluation of quality, however, in previous studies, the yield and active compounds have been considered separately. In our study, the optimal fertilizer treatment was confirmed through GRA, which considered all quality influencing factors and overall rankings. This method compensated for the gap that other studies may only partially consider or the studies that may only consider one or several factors. Similar approaches have been used in different study fields, such as chemical materials and food safety [33,34]. 

The present study showed that, compared with those of the control group (N0), the plants with fertilizer treatments showed improved parameters. These results are consistent with those obtained by predecessors on other legumes [35,36]. But interestingly, there was no significant differences in parameters between the middle treatment levels (N2 and N3), while, plants showed obvious changes between the low and high treatment levels (N1, N4, and N5). The findings of our study showed that the optimal nitrogen fertilizer levels were among the high treatment levels; similar to the findings of Cheng [28] and Song et al. [1]. The fertilizer treatments significantly influenced the fresh weight of *A. mongolica* roots, but no significant differences were observed for the aboveground plant parts compared with the control in October (Figure 2). The previous research reports that the nutrient of individual plants may be more inclined to be allocated for the growth of underground parts, or that the growth of aboveground parts is inhibited under the nitrogen stress situation [37]. That is a good explanation for our finding that the natural plant height decreased at the later stage of growth (Figure 1) because of the appearance and growing mass of seeds, which would put force on the stem to let it down to ground, and the withered stems and leaves because of the transferring of biomass to the underground parts before winter (Figure 2). Therefore, the values of above-ground indexes gradually decrease, while the values of underground indexes gradually accumulate. Under optimal partitioning theory, plants should allocate more energy and nutrients to the organ(s) that require(s) the most limiting resources [38].Therefore, in the present study, the root biomass increased sharply from August and constantly accumulated until the later harvest stage (October). 

Nitrogen fertilizer has been shown to stimulate the increase in yield of different plants [24,39]. According to the findings of the present study, the yield of *A. mongolica* among the nitrogen fertilizer levels resulted in an M-shaped fluctuating pattern. A maximum production in October was achieved by the fertilization levels of treatments N4 and N2. The optimal nitrogen fertilizer level determined in the present study differed from that of other studies [40,41], in which low levels of nitrogen fertilization were recommended. These differences in findings are presumably a result of differences in local environmental conditions. Although similar results has been reported that DW of above-ground part reaches the maximum in August and the root DW reaches the maximum in October, the differences of local precipitation and temperature lead to the difference in nitrogen fertilizer effects [40], in which the typical cold and with overcast and humid condition was reported by Qiu et al. while a continental climate of the plateau was shown in our experimental sites. Besides, the soil comes from the mixed artificial matrix [41], which was different to the natural soil in the current study, has also led to different result in nitrogen effect. Geographical and climatic factors are generally considered to be the main reasons for the differences in the yield and quality of *A. mongolica* in different production areas [42]. In fact, prior study suggested that the climatic factors of *A. mongolica* in different main producing areas have apparent effect on the active compounds. These factors are divided into two types by PCA (principal component analysis). The first class is the annual average temperature, which the active temperature above 0 °C has great contribution rate of it, and second class is the annual precipitation. The research also reveals that there is a negative correlation between the flavonoids and annual precipitation and annual average temperature. Therefore, it can be seen that *A. mongolica* prefers to grow in the areas of drought and cold condition [43]. Qinghai-Tibet plateau is renowned for its high altitude, large temperature fluctuations, long sunshine hours, and strong solar radiation. The contrasts of the findings of the present study with those of other studies may be due to the differences in growing environments in Qinghai and other regions. As mentioned in previous studies, the altitude has great effect on growth and yield [44]. In our study, the experiment conducted in high altitude area, and the annual average temperature and precipitation are lower than other producing areas in general. For example the average temperature of the Qinghai-Tibet Plateau is generally 5 °C colder than other areas at the same latitude [31]. Therefore *A. mongolica* respond to different climate conditions by regulating their secondary metabolites and other growth traits. In the further research, precipitation and temperature should be considered in the experimental design, in order to obtain the best cultivation environment on the growth and quality of *A. mongolica*.

Astragaloside IV and mullein isoflavone glucoside are the standard indicators used to evaluate the quality of *A. mongolica* and, as prescribed by the Chinese Pharmacopeia, should not be less than 0.04% and 0.02%, respectively. The content of both of these standard indicator compounds reached a maximum in October, which indicated that October is the most suitable month for *A. mongolica* harvesting. These findings are consistent with traditional agricultural experience and the findings of a previous study by Ma et al. [45]. In contrast, Wang suggested that *A. mongolica* should be harvested in spring because at this time the polysaccharide content is higher than in the autumn [46]. However, if *A. mongolica* is not harvested for the purpose of polysaccharides, it is recommended to harvest in the autumn. Because of the germination, stem pumping, and flowering of the aboveground parts of the herbs collected in spring, the content of Astragaloside IV in the root is low due to the nutrient supply to the aboveground parts. However, after October (autumn), the plant is dormant, and the nutrients are stored in the belowground parts, thus increasing the root Astragaloside IV content. Form the overall perspective, N4 is obviously higher than other nitrogen treatment levels. Equally impressive level is the N2, and the content of components under the N0 level of fertilization is not optimistic. Considering the economic benefit and costs of fertilization, the N4 (150 kg/ha) level of fertilization is recommended and plants should ideally be harvested in October. 

Another important issue is the variation in the content of active ingredients in the aboveground parts and thus further detailed assessments should be conducted to determine if the content of such compounds meet the required standards to ensure that the plants are worthwhile resources to exploit.

## 4. Materials and Methods

### 4.1. Study Area

The experiment was conducted at Minhe County, Qinghai, China (36°15′32″ N, 102°34′15″ E). The area occurs at a local average altitude of 2300 m, receives intense sun radiation, and is characterized by a typical plateau continental climate with a cool and short summer and a cold and long winter (9.38 ± 2.92 °C, January to December). The annual precipitation (41.46 ± 17.13 mm) is concentrated in summer. Climate data were obtained from a weather station (Minhe County, Qinghai Province, China, 2018). The background soil parameters and content were as follows: PH = 7.5, EC = 0.32 × 10^3^ μs/cm, organic matter = 14.63 g/kg, total nitrogen = 1.49 g/kg, total phosphorus = 0.83 g/kg, total potassium = 19.27 g/kg, and available potassium = 145 mg/kg.

### 4.2. Experimental Design

The level setting of nitrogen fertilizer is based on the soil background value. Because the N content of the soils in our experimental field is higher than that in Gansu province and other places in China, the value of our nitrogen treatments is set lower. A randomized block experiment design with five treatments and three replications was used. A blank experimental plot, i.e., with no extra applied nitrogen, was used as the control (N0). Urea was used as the nitrogen fertilizer, and five levels were applied: 37.5 kg/ha (N1), 75 kg/ha (N2), 112.5 kg/ha (N3), 150 kg/ha (N4), and 187.5 kg/ha (N5). Superphosphate was used as the phosphate fertilizer with 150 kg/ha and potassium sulphate was used as potassium fertilizer with 112.5 kg/ha. All of the fertilizer was applied before the transplant. There was a regular practice of pulling weeds, but without irrigation. 

The experimental field included 16 plots (*n* = 15 experimental plots, *n* = 1 control plot), each covering an area of 8.4 m^2^. Seedlings were transplanted 20 cm apart and in rows spaced 15 cm apart. Plants were cultivated at the end of April and harvested in late August, September, and October, during which, 20 plants were randomly selected for harvesting. After cleaning, the roots and aboveground parts were separated and weighed to determine the fresh weight with an electronic balance. After sun drying [4], the parts were reweighed (dry weight) and then the root ground into powder for use in the chemical analysis. In addition, the determination of the root length was with a tape measure and the determination of the root diameter was with a Vernier caliper.

### 4.3. Plant Material

*Astragalus mongolica* was used as the plant material. The life cycle of this species usually lasts 2 years. First year seedlings were collected from Minxian County, Gansu, China, identified by Professor Guoying Zhou, and transplanted the following spring. The harvest time of this species is autumn of the second year. Healthy seedlings were selected at the transplanting stage to eliminate weak seedlings.

### 4.4. Preparation of Sample Solution and Standard Solution

Powdered samples were weighed accurately to 1.0 g, and dissolved in 40 mL methyl alcohol. The flasks were then heated to reflux for 3 h. After cooling to room temperature (<20 °C), the samples were filtered and evaporated. The final concentrate was then diluted with methyl alcohol (up to 10 mL volume) and filtered through a 0.45 μm organic membrane. Samples solutions of flavones were then subjected to HPLC analysis [1]. 

Dried samples were weighed accurately to 1.0 g, and dissolved in 30 mL methyl alcohol. Samples underwent ultrasonic extraction for 30 min and subsequent filtration. Filtrates were collected. The resulting residues were dissolved in 30 mL methyl alcohol, subjected to ultrasonic extraction for another 30 min, and then filtered. The two filtrates per sample were merged and then evaporated. The residues were dissolved in 25 mL concentrated ammonia water and shaken occasionally for 10 min. The mixtures were then saturated with *n*-butanol (30 mL and 20 mL) and extracted twice after being combined with extraction solution and dried by rotary evaporation at 65 °C. The residues were dissolved in and fixed in methanol (10 mL) and shaken well. After filtering through a 0.45 μm organic membrane, the solutions of Astragaloside IV were ready for HPLC analysis [1]. The injection volume was 10 μL for all samples.

Standard solutions of mullein isoflavone glucoside (0.81 mg/mL), ononin (0.37 mg/mL), mullein isoflavone (0.16 mg/mL), formononetin (0.13 mg/mL), and Astragaloside IV (1.67 mg/mL) were made by dissolving the chemicals in methanol. The standard solutions were injected in volumes of 2, 5, 10, 15, 20, 25, 30, 35, 40, and 50 µL into the HPLC system for calibration.

The standards for mullein isoflavone glucoside, ononin, mullein isoflavone, formononetin, and astragaloside IV used in this study were purchased from Sigma (USA). Ultrapure water was obtained from a Millipore Milli-Q system (Bedford, MA, USA). The HPLC chromatographic grade methanol and ethanol were provided by the YuWang Group (Shandong, China). Other chemicals used were of analytical grade.

### 4.5. Chromatographic Conditions

Analyses were performed using an Agilent 1260 (Agilent USA) Infinity II Quaternary Pump (G7111A). The detectors were G7114A DAD and ELSD, the autosampler was G7129A, and Agilent HPLC software was used (Germany). Chromatography columns Agilent 5 HC-C18 (4.6 × 250.0 mm, 5 μm) and Agilent EC-C18 (150.0 × 4.6 mm, 4 μm) were used to analyze the flavones and Astragaloside IV, respectively, at a wavelength of 260 nm. 

The flavone condition of mobile phase A was 0.01% *v*/*v* phosphoric acid in ultrapure water and mobile phase B was acetonitrile. The flow rate was 1.0 mL/min and the column temperature was 30 °C. The gradient elution of flavonoids is shown in Table 1. The Astragaloside IV condition of mobile phase A was acetonitrile (40%) and mobile phase B was ultrapure water (60%). The flow rate was 1.2 mL/min and the column temperature was 30 °C. Peaks appeared within 5 min of the analyses.

Three different chromatographic columns were used to examine the optimal conditions for flavonoids and three different flow rates were used in the same column to test the optimal conditions for Astragaloside IV. According to the comprehensive evaluation, the Agilent 5 HC-C18 and the Agilent EC-C18 columns were selected to determine the flavonoids and Astragaloside IV, respectively, with a flow rate of 1.2 mL/min, since they provided the best chromatograms.

### 4.6. Grey Relation Analysis

#### 4.6.1. Establishment of the Data Set

The yield and content of mullein isoflavone glucoside, Astragaloside IV, ononin, calycosin, and formononetin were used as evaluation indexes to form evaluation unit sequences. The resulting data set is shown in Table S1. The evaluation unit sequence was {Xij} (i = 1, 2, ..., n; j = 1, 2, ..., m) and the GRA was used as the evaluation method. The reference sequence was first determined and then the optimal reference sequence and the worst reference sequence were set as {Xsj} and {Xtj}, respectively.

#### 4.6.2. Dimensionless Processing of Original Data

The issue of different measures among evaluation indexes requires the normalization of the original data. The value normalized is that this value divided by the mean value of the index in N0-N5. The normalization formula was: Yij = Xij/‘Xj, where Yij is the dimensionless data, Xij is the original data, and Xj is the mean value of the j index of n samples. The dimensionless data are shown in Table S2.

#### 4.6.3. Calculation of the Correlation Coefficients

The correlation coefficients relative to the optimal reference sequences were calculated as follows:

(1) $$\int_{j}^{i}(s)\;=\;\frac{\Delta \min\;+\;\rho \Delta \max\;}{\left|\;\mathrm{Yij}\;\text{–}\mathrm{Ysj}\;\right|\;+\;\rho \Delta \max}$$

where s is the mean optimal reference sequences data; Δmin = min|Yij − Ysj|; Δmax = max|Yij − Ysj|; i = 1,2,...n; j = 1,2,...m; and ρ is the resolution coefficient, which is usually 0.5.

The correlation coefficients relative to the worst reference sequences were calculated as follows:

(2) $$\int_{j}^{i}(t)\;=\;\frac{\Delta \min\;+\;\rho \Delta \max\;}{\left|\;\mathrm{Yij}\;\text{–}\mathrm{Ytj}\;\right|\;+\;\rho \Delta \max}$$

where t is the mean worst reference sequences data; Δmin = min|Yij − Ytj|; Δmax = max|Yij − Ytj|; i = 1,2,...n; j = 1, 2,...m; and ρ is the resolution coefficient, which is usually 0.5.

#### 4.6.4. Calculation of the Correlation Degrees

The correlation degrees relative to the optimal reference sequences (s) were calculated as follows:

(3) $$\mathrm{Ri}\left(s\right)\;=\;\frac{1}{m}\overset{m}{\underset{j\;=\;1}{\sum }}\int_{j}^{i}\left(s\right)$$

and the correlation degrees relative to the worst reference sequences (t) were calculated as

(4) $$\mathrm{Ri}\left(t\right)\;=\;\frac{1}{m}\overset{m}{\underset{j\;=\;1}{\sum }}\int_{j}^{i}\left(t\right)$$

#### 4.6.5. Definition and Calculation of the Relative Correlations

The relative correlations were calculated as follows:

(5) $$\mathrm{Ri}\;=\;\frac{\mathrm{Ri}(s)}{\mathrm{Ri}\left(s\right)\;+\;\mathrm{Ri}(t)}$$

where the larger the Ri (s) is, the greater the correlation degree between the evaluation unit sequence and the optimal reference sequence. The smaller the Ri (t) is, the greater the correlation degree between the evaluation unit sequence and the worst reference sequence. The ideal optimal evaluation unit will have the largest correlation with the optimal reference sequence and the smallest correlation with the worst reference sequence. The larger the Ri is, the better the evaluation unit is. The rank of the evaluation sequence, according to the degree of relative correlation, is shown in Table 2.

### 4.7. Data Analysis

The data were analyzed with one-way ANOVA using SPSS 22.0 (IBM Corporation, Armonk, NY, USA). Duncan’s multiple range tests were used to assess the data when the differences between means were significant (*p* < 0.05). Figures were drawn using Origin 2018 (Northampton, MA, USA). Excel was used to conduct the GRA.

## 5. Conclusions

The experiment was conducted in the Qinghai province. Five active compounds and various growth parameters of *A. mongolica* were measured over three months. A GRA was used to evaluate the quality of the medicinal compounds. The N4 level of nitrogen fertilizer was determined to be best, not only with regards to promoting the chemical components but also the yield of *A. mongolica*. The content of Astragaloside IV and mullein isoflavone glucoside had already meet the standard of Chinese Pharmacopoeia in September and their content of October was higher than that of previous two month. However, there was no obvious influence on the content in the September and October. The content of ononin, calycosin, and for mononetin differed significantly among the different months. In addition, nitrogen fertilizer also had an apparent effect on the root and aboveground biomass.

## Figures and Tables

**Table 1:** Conditions of linear gradient elutions for flavonoids found in *Astragalus mongolica.*

Time (min)	Mobile Phase A	Mobile Phase B
0~5	10	90
5~15	18	82
15~25	20	80
25~32	25	75
32~52	30	70

**Table 2:** Comprehensive quality evaluation results of *Astragalus mongolica* under different nitrogen fertilizer levels: 37.5 kg/ha (N1), 75 kg/ha (N2), 112.5 kg/ha (N3), 150 kg/ha (N4), and 187.5 kg/ha (N5).

Treatment	Relative Correlation Degree	Rank
N0	0.2984	6
N1	0.4775	4
N2	0.5244	3
N3	0.4529	5
N4	0.5684	1
N5	0.5428	2

## Supplementary Materials

The following are available online. Table S1: The data of yield and active components of *Astragalus mongolica* under different nitrogen fertilizer levels: 37.5 kg/ha (N1), 75 kg/ha (N2), 112.5 kg/ha (N3), 150 kg/ha (N4), and 187.5 kg/ha (N5). Table S2: Standardization of the data of *Astragalus mongolica* yield and active components under different nitrogen fertilizer levels: 37.5 kg/ha (N1), 75 kg/ha (N2), 112.5 kg/ha (N3), 150 kg/ha (N4), and 187.5 kg/ha (N5). Table S3: Relation coefficients and degrees of evaluation units relative to the best evaluation sequence for yield and active components data of *Astragalus mongolica* under different nitrogen fertilizer levels: 37.5 kg/ha (N1), 75 kg/ha (N2), 112.5 kg/ha (N3), 150 kg/ha (N4), and 187.5 kg/ha (N5). Table S4: Relation coefficients and degrees of evaluation units relative to the worst evaluation sequence of yield and active components data of *Astragalus mongolica* under different nitrogen fertilizer levels: 37.5 kg/ha (N1), 75 kg/ha (N2), 112.5 kg/ha (N3), 150 kg/ha (N4), and 187.5 kg/ha (N5).
