# Supplementary material for: Nitrogen Fertilizer Levels Affect the Growth and Quality Parameters of Astragalus mongolica
Source: Molecules. 2020 Jan 16;25(2):381. doi: 10.3390/molecules25020381 (PMC7024162; doi:10.3390/molecules25020381)
Supplement: Supplementary file 1 [file molecules-25-00381-s001.pdf]

Table S1. The data of yield and active components of *Astragalus mongolica* under different nitrogen fertilizer levels: 37.5 kg/ha (N1), 75 kg/ha (N2), 112.5 kg/ha (N3), 150 kg/ha (N4), and 187.5 kg/ha (N5).

Treatment	Yield (kg/ha)	Mullein isoflavone glucoside (%)	Astragaloside IV (%)	Ononin (%)	Calycosin (%)	Formononetin (%)
N0	9473.80	0.0454	0.1436	0.0354	0.0066	1.06E-05
N1	13067.50	0.038	0.1466	0.0414	0.0085	0.000135
N2	9119.72	0.0439	0.1678	0.0455	0.0088	0.000141
N3	9405.43	0.0433	0.1514	0.0394	0.0078	0.000169
N4	14925.19	0.038	0.1329	0.0499	0.0099	0.000146
N5	9812.80	0.0538	0.1533	0.044	0.0085	0.000103
Mean	10967.41	0.0437	0.1493	0.0426	0.0083	0.000118
Best reference sequence	14925.19	0.0538	0.1678	0.0499	0.0099	0.000169
Worst reference sequence	9119.72	0.038	0.1329	0.0354	0.0066	1.06E-05

Table S2. Standardization of the data of *Astragalus mongolica* yield and active components under different nitrogen fertilizer levels: 37.5 kg/ha (N1), 75 kg/ha (N2), 112.5 kg/ha (N3), 150 kg/ha (N4), and 187.5 kg/ha (N5).

Treatment	Yield (kg/ha)	Mullein isoflavone glucoside (%)	Astragaloside IV (%)	Ononin (%)	Calycosin (%)	Formononetin (%)
N0	0.8638	1.0389	0.9618	0.8310	0.7952	0.0898
N1	1.1915	0.8696	0.9819	0.9718	1.0241	1.1441
N2	0.8315	1.0046	1.1239	1.0681	1.0602	1.1949
N3	0.8576	0.9908	1.0141	0.9249	0.9398	1.4322
N4	1.3609	0.8696	0.8902	1.1714	1.1928	1.2373
N5	0.8947	1.2311	1.0268	1.0329	1.0241	0.8729
Mean	1.0000	1.0000	1.0000	1.0000	1.0000	1.0000
Best reference sequence	1.3609	1.2311	1.1239	1.1714	1.1928	1.4322
Worst reference sequence	0.8315	0.8696	0.8902	0.8310	0.7952	0.0898

Table S3. Relation coefficients and degrees of evaluation units relative to the best evaluation sequence for yield and active components data of *Astragalus mongolica* under different nitrogen fertilizer levels: 37.5 kg/ha (N1), 75 kg/ha (N2), 112.5 kg/ha (N3), 150 kg/ha (N4), and 187.5 kg/ha (N5).

Treatment	Yield (kg/ha)	Mullein isoflavone glucoside (%)	Astragaloside IV (%)	Ononin (%)	Calycosin (%)	Formononetin (%)	Relevancy degree
N0	0.3475	0.4844	0.4196	0.3333	0.3333	0.3333	0.3753
N1	0.6098	0.3333	0.4514	0.4608	0.5407	0.6977	0.5156
N2	0.3333	0.4433	1.0000	0.6238	0.5952	0.7333	0.6215
N3	0.3446	0.4302	0.5163	0.4084	0.4381	1.0000	0.523
N4	1.0000	0.3336	0.3333	1.0000	1.0000	0.7705	0.7396
N5	0.3622	1.0000	0.5465	0.5510	0.5395	0.5459	0.5908

Table S4. Relation coefficients and degrees of evaluation units relative to the worst evaluation sequence of yield and active components data of *Astragalus mongolica* under different nitrogen

fertilizer levels: 37.5 kg/ha (N1), 75 kg/ha (N2), 112.5 kg/ha (N3), 150 kg/ha (N4), and 187.5 kg/ha (N5).

Treatment	Yield (kg/ha)	Mullein isoflavone glucoside (%)	Astragaloside IV (%)	Ononin (%)	Calycosin (%)	Formononetin (%)	Relevancy degree
N0	0.8913	0.5166	0.6185	1.0000	1.0000	1.0000	0.8377
N1	0.4237	1.0000	0.5603	0.5465	0.4650	0.3896	0.5642
N2	1.0000	0.5733	0.3333	0.4172	0.4311	0.3793	0.5224
N3	0.9104	0.5968	0.4847	0.6445	0.5823	0.3333	0.5920
N4	0.3333	0.9972	1.0000	0.3333	0.3333	0.3701	0.5612
N5	0.8073	0.3333	0.4608	0.4576	0.4659	0.4612	0.4977
